# Differentiation of human induced pluripotent stem cells into nucleus pulposus-like cells

**DOI:** 10.1186/s13287-018-0797-1

**Published:** 2018-03-09

**Authors:** Ruhang Tang, Liufang Jing, Vincent P. Willard, Chia-lung Wu, Farshid Guilak, Jun Chen, Lori A. Setton

**Affiliations:** 1 Department of Orthopaedic Surgery, Washington University, St. Louis, MO USA; 2 Shriners Hospitals for Children—St. Louis, St. Louis, MO USA; 3 Department of Biomedical Engineering, Washington University, 1 Brookings Drive, St. Louis, MO 63130 USA; 4 Cytex Therapeutics, Inc., Durham, NC USA; 5 Department of Orthopaedic Surgery, Duke University, Durham, NC USA

**Keywords:** Human induced pluripotent stem cells, Intervertebral disc, Degenerative disc disease, Nucleus pulposus, Directed differentiation, Chondrocyte, Cartilage

## Abstract

**Background:**

Intervertebral disc (IVD) degeneration is characterized by an early decrease in cellularity of the nucleus pulposus (NP) region, and associated extracellular matrix changes, reduced hydration, and progressive degeneration. Cell-based IVD therapy has emerged as an area of great interest, with studies reporting regenerative potential for many cell sources, including autologous or allogeneic chondrocytes, primary IVD cells, and stem cells. Few approaches, however, have clear strategies to promote the NP phenotype, in part due to a limited knowledge of the defined markers and differentiation protocols for this lineage. Here, we developed a new protocol for the efficient differentiation of human induced pluripotent stem cells (hiPSCs) into NP-like cells *in vitro*. This differentiation strategy derives from our knowledge of the embryonic notochordal lineage of NP cells as well as strategies used to support healthy NP cell phenotypes for primary cells in vitro.

**Methods:**

An NP-genic phenotype of hiPSCs was promoted in undifferentiated hiPSCs using a stepwise, directed differentiation toward mesodermal, and subsequently notochordal, lineages via chemically defined medium and growth factor supplementation. Fluorescent cell imaging was used to test for pluripotency markers in undifferentiated cells. RT-PCR was used to test for potential cell lineages at the early stage of differentiation. Cells were checked for NP differentiation using immunohistochemistry and histological staining at the end of differentiation. To enrich notochordal progenitor cells, hiPSCs were transduced using lentivirus containing reporter constructs for transcription factor brachyury (T) promoter and green fluorescent protein (GFP) fluorescence, and then sorted on T expression based on GFP intensity by flow cytometry.

**Results:**

Periods of pellet culture following initial induction were shown to promote the vacuolated NP cell morphology and NP surface marker expression, including CD24, LMα5, and Basp1. Enrichment of brachyury (T) positive cells using fluorescence-activated cell sorting was shown to further enhance the differentiation efficiency of NP-like cells.

**Conclusions:**

The ability to efficiently differentiate human iPSCs toward NP-like cells may provide insights into the processes of NP cell differentiation and provide a cell source for the development of new therapies for IVD diseases.

**Electronic supplementary material:**

The online version of this article (10.1186/s13287-018-0797-1) contains supplementary material, which is available to authorized users.

## Background

Intervertebral disc (IVD) disorders may contribute to back pain and disability affecting over 80% of adults and responsible for a socioeconomic toll of nearly $100 billion annually in the United States alone [1–4]. The IVD is a complex structure formed by the peripheral anulus fibrosus, which circumferentially encloses the nucleus pulposus (NP), and the superior and inferior cartilage endplates. The NP is derived from mid-mesoderm notochordal structures [5–10], and is composed mainly of water, proteoglycans, collagen type II, and morphologically distinct cells that are responsible for homeostasis of the NP matrix [11–14]. The NP has been reported as the first structure to be affected by age-associated IVD degeneration [15], with decreased cellularity and water content, loss of proteoglycans in the extracellular matrix, and increased matrix stiffness [16]. Aging leads to loss of the juvenile NP cell population, that is derived from the embryonic notochord and remains distinct for cells of larger size with a highly vacuolated cell structure [17, 18]. The early decrease in NP cellularity is believed to be a key factor contributing to the progressive nature of IVD pathology, with the disc being unable to restore healthy matrix, hydration, or composition due to the absence of phenotypically appropriate cells.

There is a widespread need to develop early interventions to arrest IVD pathology progression, with great interest in cell supplementation approaches that can maintain matrix synthesis and restore a young, nondegenerate, functional disc structure. The therapeutic potential of cell delivery to pathological herniated or degenerated IVD has been recognized for promoting regeneration [19–21]. However, limited accessibility of human cell sources has been a major hurdle for cell-based therapy for IVD degeneration. In this regard, we and others have previously demonstrated that human umbilical cord mesenchymal stromal cells, as well as human and mouse induced pluripotent stem cells (iPSCs), were capable of expressing markers of NP cells when cultured upon defined substrates and with defined culture media [22–27]. In particular, human iPSCs (hiPSCs) hold great cell therapeutic potential due to their pluripotency, high proliferation rate, and patient specificity. The goal of this study was to develop a robust set of working protocols for differentiating hiPSCs to an NP cell lineage using physically and chemically defined conditions. A directed, stepwise differentiation protocol was developed to generate NP-like cells through an early inductive stage of mesodermal and then notochordal lineages; followed by cell expansion and pellet culture stages. To further enrich the desired cell population, a subset of cells was sorted by flow cytometry using a green fluorescent protein (GFP) reporter for the transcription factor brachyury (T). The expression of markers of NP-like cells at the end of differentiation protocol was determined by measures of cell morphology, immunohistochemistry, and histology.

## Methods

### Human induced pluripotent stem cells and visualization of pluripotency markers

The hiPSC cell line was obtained from the Duke iPSC Shared Resource Facility; this cell line had been generated as a clone from human neonatal foreskin fibroblasts by reprogramming with a set of episomal plasmids (A14703; Invitrogen, Carlsbad, CA, USA) delivering the reprogramming genes *Oct4*, *Sox2*, *Nanog*, *Lin28*, *L-Myc*, *Klf4*, and *SV40LT*. The resulting hiPSC line was propagated as colonies on vitronectin-coated dishes using Essential 8™ medium (E8 medium; STEMCELL Technologies, Seattle, WA, USA), a serum-free, xeno-free medium that supports feeder-free culture conditions for iPSCs.

The expression of pluripotent markers in iPSC colonies was evaluated by immunofluorescence. Briefly, hiPSCs were cultured on a vitronectin-coated chamber slide and allowed to form into cell colonies in E8 medium. The cell chamber was washed with PBS and fixed using paraformaldehyde (4% in PBS; Electron Microscopy Sciences, Hatfield, PA, USA). Cells were stained for DNA using SYTO Green, and for pluripotent markers as follows. The pluripotency testing kit with polyclonal antibodies (Applied StemCell, Milpitas, CA, USA) was used for verifying positive expression of pluripotent markers included anti-OCT4, anti-SOX2, anti-SSEA-4, anti-TRA1–60, and anti-TRA1–81. Secondary antibodies for detection were all conjugated to appropriate Alexa fluorochromes (Molecular Probes, Eugene, OR, USA). Fluorescent cell imaging was performed on a Zeiss 510 inverted confocal microscope (Carl Zeiss, Thornwood, NY, USA) with Zeiss AIM LSM Image browser.

### Media supplementation effects on hiPSC expression of mesodermal and notochordal markers

To test for an effect of media supplementation on expression of transcription factors potentially related to NP-like cell differentiation, RT-PCR was used to evaluate mesodermal/notochordal markers in hiPSCs cultured in cell colonies with two groups of supplemented media, as compared to basal media (TeSR™-E6 media; STEMCELL Technologies). First, hiPSCs were cultured in vitronectin-coated six-well plates (~ 20 colonies/well) with basal medium as the control, or in basal medium supplemented with FGF2 (20 ng/ml; R&D Systems, Minneapolis, MN, USA) and BMP4 (40 ng/ml; R&D) for up to 5 days (supplemented group). Separately, hiPSCs were cultured in basal medium supplemented with FGF2, BMP4, Wnt-3a (25 ng/ml; R&D), and Activin A (50 ng/ml; R&D) and tested for induction of notochordal markers by measuring *NOTO* over 5 days (supplemented “plus” group). At the end of culture, cells were evaluated for transient changes in expression of key mesodermal markers (*T, MIXL1, CDX2*) and node/notochord markers (*FOXA2, SHH, NOG*, and *NOTO*) via RT-PCR, as follows. Total RNA was isolated from cells (1–5 days) using column purification with the RNAeasy kit plus DNase I digestion (Qiagen, Valencia, CA, USA) and then transcribed to cDNA using SuperScript VILO (Invitrogen). Quantitative PCR was performed for each transcription factor of interest using Taqman primer/probe sets (Applied Biosystems, Foster City, CA, USA) (a list of primer/probe sets is presented in Additional file 1: Table S1) with 18S ribosomal RNA (*rRNA*) as a housekeeping gene. Relative gene expression differences were quantified amongst cells in the control, supplemented, and supplemented “plus” groups using the comparative Ct method. Statistical analyses were performed to detect a difference in ΔCt (Ct of target minus Ct of 18 s rRNA) values between the control and each treatment group for each target gene using a one-factor ANOVA (StatView; SAS Institute, Cary, NC, USA). Fold-differences in relative mRNA levels (2^–∆∆Ct^) between the basal control media and each supplemented media group were reported if greater than or equal to 2, and where ANOVA detected a difference at *p* < 0.05.

### Multistep differentiation of hiPSCs toward NP-like cells (NP differentiation media)

**Fig. 1:**
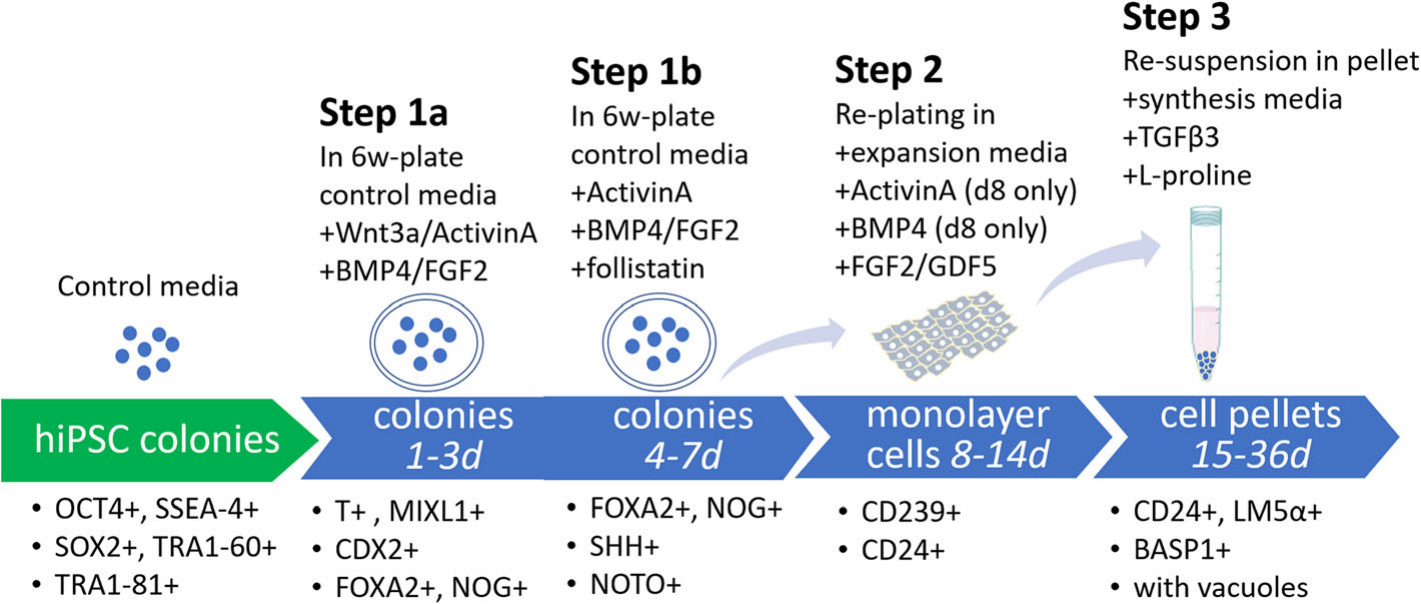
Protocol design. Schematic diagram illustrating three steps for directed differentiation of undifferentiated hiPSCs into NP-like cells. E8 medium = Essential 8™ medium (STEMCELL Technologies, Seattle, WA, USA); hiPSC = human induced pluripotent stem cell; d = days; 6w = six-well; SHH = sonic hedgehog; T = brachyury; BMP4 = bone morphogenetic protein 4; FOXA2 = forkhead box protein A2; FGF2 = basic fibroblast growth factor; CD24 = cluster of differentiation 24 protein; BASP1 = brain abundant membrane attached signal protein 1; MIXL1 = paired-type homeobox transcription factor identified in human; CDX2 = member of the caudal-related homeobox transcription factor family; TGF = transforming growth factor; LMα5 = alpha-5 subunit of heterotrimeric laminin; NOG = noggin; NOTO = notochord homeobox

A directed, stepwise differentiation protocol was developed to generate NP-like cells through notochordal then mesodermal lineages based on prior studies [28–32] (Fig. 1 and Table 1). A temporal protocol was determined for media supplementation that we proposed would promote expression of notochordal/mesodermal-related markers in advance of NP-directed cell differentiation in three steps. The detailed protocol was defined as shown in Fig. 1 and Table 1 and is dependent on a set of media conditions termed NP differentiation media (NPDM). NPDM was defined by factors added to cell culture to promote differentiation of iPSCs into NP-like cells. The medium was changed daily until pellet formation, and thereafter was changed every other day.

**Table 1:** Nucleus pulposus differentiation media defined by factors added to cell culture to promote differentiation of iPSCs into NP-like cells in a directed, stepwise differentiation protocol as illustrated in Fig. 1. Culture medium was changed daily until pellet formation. Pellet medium was changed every other day

Stage	Day	Wnt-3a (ng/ml)	ActivinA (ng/ml)	FGF2 (ng/ml)	BMP4 (ng/ml)	Follistatin (ng/ml)	GDF5 (ng/ml)	TGF-β3 (ng/ml)
Step 1a: notochord/mesoderm differentiation	1	25	50	–	–	–	–	–
	2	25	25	20	40	–	–	–
	3	25	10	20	40	–	–	–
Step 1b: notochord/mesoderm differentiation	4	–	5	20	40	100	–	–
	5	–	5	20	40	100	–	–
	6	–	2	20	40	100	–	–
	7	–	2	20	40	100	–	–
Step 2: expansion in monolayer and differentiation	Replate 8	–	2	20	20	–	–	–
	9–14	–	–	5	–	–	40	–
Step 3: pellet culture and differentiation	15–36	–	–	–	–	–	–	10

Protocol illustrated in Fig. 1. Culture medium was changed daily until pellet formation. Pellet medium was changed every other day

BMP bone morphogenetic protein 4, iPSC induced pluripotent stem cell, FGF2 basic fibroblast growth factor, NP nucleus pulposus, TGF transforming growth factor

The stepwise differentiation protocol has the following three steps: (step 1a/b) early growth factor-induced differentiation (in colonies); (step 2) cell expansion (in monolayer); and (step 3) cell pellet formation. In step 1, cells were cultured as colonies in vitronectin-coated six-well plates (~ 20 colonies/well) with basal media that was supplemented as described over a 7-day period. In this initial step, cell differentiation was induced by first adding Wnt-3a and Activin A to TeSR™-E6 media from days 1 to 3, FGF2 and BMP4 from days 2 to 7, and then Activin A and follistatin (Sigma, St. Louis MO, USA) from days 4 to 7; all doses of supplements are as presented in Table 1. The sequential reduced concentration of Activin A and follistatin were used to inhibit the expression of endodermal lineage genes [28] during this initial differentiation step. As a control, cells were cultured for the first 7 days in basal media without any growth factor supplementation.

In step 2, cells were detached and dissociated using 0.025% trypsin/EDTA (Lonza, Walkersville, MD, USA) and replated upon 0.1% gelatin-coated T75 flasks at a density of 50,000 cells/cm^2^ in expansion media. The expansion media consisted of DMEM-HG (Life Technologies, Grand Island, NY, USA), 10% FBS (Atlanta Biologicals, Lawrenceville, GA, USA), 1% ITS+, 1% NEAA, and 1% penicillin/streptomycin (all from Life Technologies); expansion media were also supplemented with 2 ng/ml Activin A, 20 ng/ml FGF2, and 20 ng/ml BMP-4 for the first day, and then replaced with only 5 ng/ml FGF2 (R&D) and 40 ng/ml GDF5 (R&D) for an additional 6 days of cell expansion. Media were exchanged as presented in Table 1. As a control, replated cells were cultured for the additional 7 days in expansion medium without growth factor supplementation.

In step 3, at the end of monolayer culture (~ 90% confluence, 7 days), cells were used to form pellets (200,000 cells/pellet) in 15-ml conical tubes. Pellets were cultured in a serum-free media to promote matrix synthesis that contained DMEM-HG, 1% ITS+, 1% NEAA, 1% penicillin/streptomycin, 50 μg/ml ascorbic acid-2-phosphate (Sigma), 40 μg/ml l-proline (Sigma), and 10 ng/ml TGF-β3 (R&D). Since consensus has not been reached on media supportive of the NP cell phenotype, this choice for serum-free media was chosen based on an adaptation of media shown to be NP-genic in our prior study on mouse iPSCs [22]; the addition of l-proline and TGF-β3 was used to be consistent with conditions that promote maximal proteoglycan and collagen production. As a control, pellets were cultured for the additional 21 days in the same medium but without growth factor supplementation. Pellets were maintained in culture for 21 days at 5% CO_2_ and 20% O_2_, with media changes every other day.

### Histology and immunohistochemistry

Pellets were embedded in Optimal Cutting Temperature (OCT) compound (Sakura Finetek, Torrance, CA, USA), flash-frozen in liquid nitrogen, and stored at −80 °C until cryosectioning. Cell morphology and proteoglycan synthesis were assessed by histological staining using H&E and Safranin O [33]. Following previously established protocols, 7-μm-thick sections were fixed and incubated with specific human antibodies for NP markers: CD24 (551,133; BD), LMα5 (MAP1924; EMD Millipore), CD239 (Epitomics 2994, Burlingame CA USA), and BASP1 (AB9306; Millipore) [33, 34]. All sections were incubated with appropriate secondary antibodies (Alexa Fluor 488; Molecular Probes) for 30 min in blocking solution, counterstained with propidium iodide (0.2 mg/ml; Sigma) to label cell nuclei, and imaged using confocal laser scanning microscopy (Zeiss LSM 510).

### Flow cytometry analysis of notochordal transcription factor brachyury expression via the T-fluorescent reporter construct

A separate experiment was performed to enrich notochordal progenitor cells from hiPSCs to test their preferred utility in generating NP cells. hiPSCs were transduced using lentivirus containing reporter constructs for the transcription factor brachyury (T) promoter and GFP fluorescence reporter (eGFP/Rex-Neo^r^, Addgene Plasmid 21,222). The plasmids contained a Neo resistance gene to facilitate selection. Stably transfected cells were selected by culture with 10 mg/ml of Geneticin (G418; Invitrogen) for 4 days. This reporter cell line was separately maintained in culture in a similar feeder-free, serum-free, xeno-free culture condition as already described and in vitronectin-coated dishes with E8 medium. Intracellular expression of transcription factor brachyury (T) in cells was tested by both brachyury–promoter–GFP fluorescence and immunofluorescence after cells were induced to differentiate by FGF2 (20 ng/ml) and BMP4 (40 ng/ml) for 3 days on vitronectin coated six-well plates. Treated cells were incubated with monoclonal antibodies against brachyury (T) (sc-20,109; Santa Cruz Biotechnology, Dallas, TX, USA) and appropriate isotype controls, followed by incubation with fluorescently labeled secondary antibodies. Cells were analyzed by flow cytometry (Model C6; Accuri, Ann Arbor, MI, USA) to determine the percentage of cells positive for T or GFP (% cells). Flow cytometry analysis was repeated for two sets of cell preparations, and the average of values for percentage of positive cells was reported.

### NP differentiation from a selected set of notochordal progenitor cells

Transduced hiPSCs were cultured as colonies in vitronectin-coated six-well plates with supplemented media as described for step 1a of the NPDM protocol for 3 days (Fig. 1 and Table 1). At the end of 3 days of differentiation, cells were sorted into two fractions of cells (GFP^+^ vs GFP^–^) based on GFP intensity (BD FACSAria II, Special Order System). Sorted cells in both GFP^+^ and GFP^–^ were cultured overnight and then promoted to continue differentiation, expansion, and pellet formation by stepping through protocol steps 1b–3 of the NPDM differentiation protocol described earlier (Fig. 1 and Table 1). At the end of the culture, cells were used for histology and immunolabeling.

For immunocytofluorescence, cells were cultured first in monolayer as already described and allowed to differentiate for 12 days as described in step 1b and step 2 of the NPDM protocol (Fig. 1 and Table 1). At the end of this period and prior to pellet formation, cells were fixed by 4% paraformaldehyde (10 min) and incubated with mouse anti-human CD24 monoclonal antibody (1:100, 555426, 2 h; BD) followed by goat anti-mouse IgG antibody Alexa Fluor 488 (R37120, 1 h; ThermoFisher Scientific). The same cell preparations were washed three times in PBS, incubated with rabbit anti-CD-239 monoclonal antibody (1:150, 2994, 1 h; Epitomics), and then labeled with chicken anti-rabbit IgG secondary antibody (Alexa Fluor 647, A21443, 30 min; Invitrogen). All cells were counter-stained with DAPI (Sigma) to visualize cell nuclei.

## Results

### Characterization of human iPSCs

**Fig. 2:**
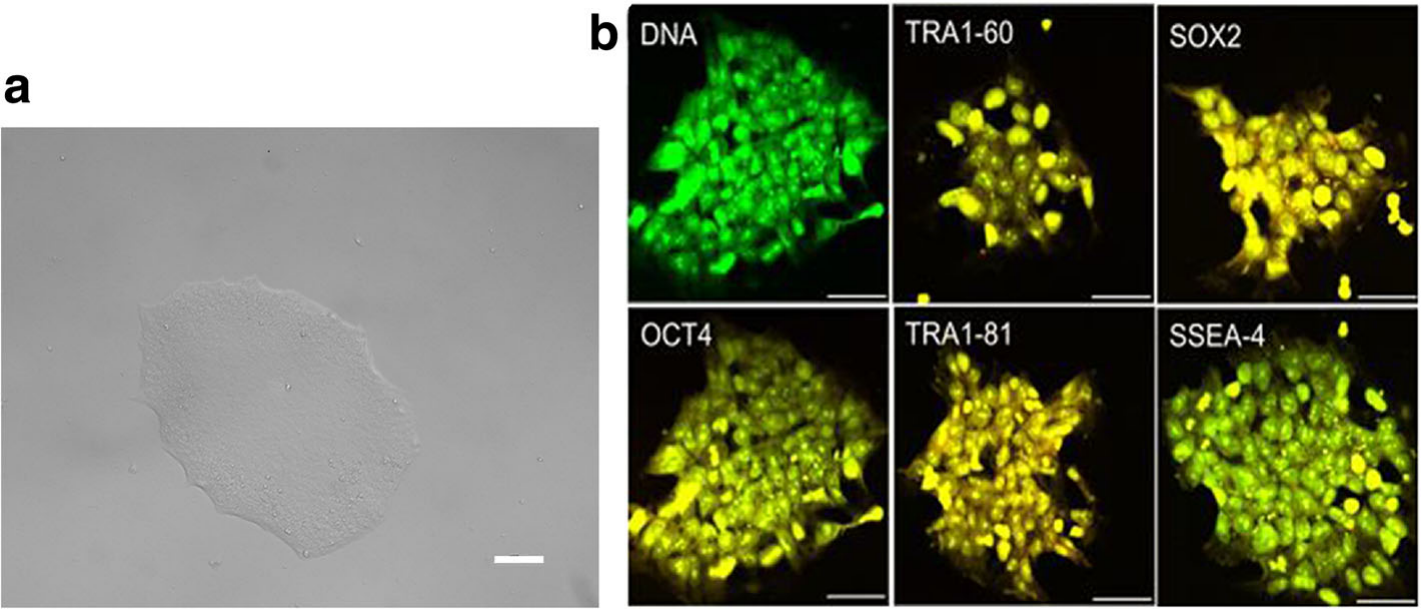
Pluripotency for derived colonies confirmed via immunofluorescence. **a** Undifferentiated human iPSCs cultured in colonies on vitronectin-coated plates until induction of differentiation (scale bar = 50 μm). **b** Immunofluorescence showing DNA (green) and positive pluripotent marker expression (red) of TRA1–60, SOX2, OCT4, TRA1–81, and SSEA-4. Yellow indicates costaining of green nuclei and red pluripotent markers (scale bar = 50 μm)

Successful reprogramming and pluripotency was verified by appropriate morphology and size of the cell colony (Fig. 2a) and the presence of pluripotency markers via immunofluorescence: for OCT4, SOX2, SSEA-4, TRA1–60, and TRA1–81 (Fig. 2b). As we used polyclonal antibodies for this immunohistochemistry, appropriate IgG controls were not used and negative expression was determined with a human foreskin fibroblast cell line as a control (data not shown).

### Early mesoderm and notochord markers are induced by BMP4/FGF2 and Wnt-3a/Activin A

**Fig. 3:**
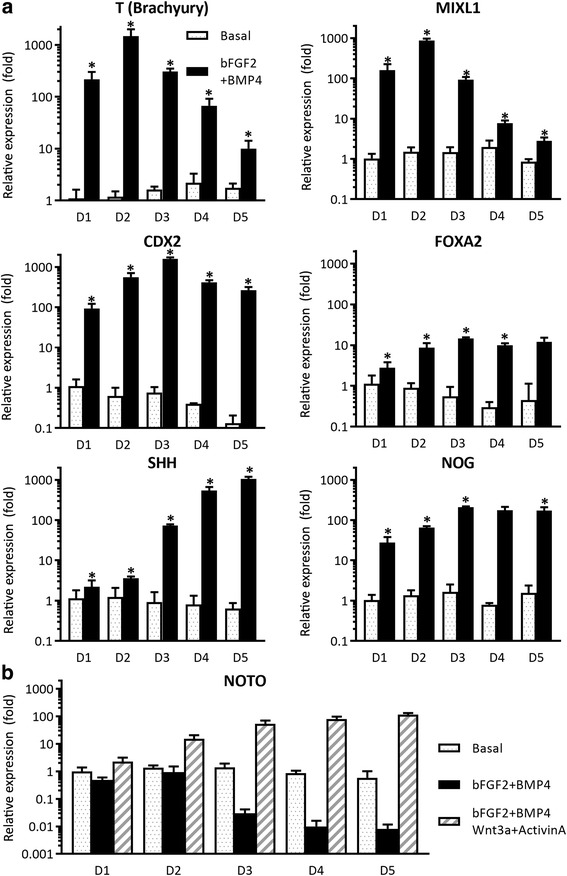
Media supplements added to hiPSC colonies to promote differentiation of mesodermal lineage. **a** In comparison to culture in basal conditions, RT-PCR demonstrated higher mRNA levels for brachyury (*T*) at day 2 (D2), along with similarly high mRNA levels for the mesoderm markers *MIXL1* and *CDX2* (ANOVA, *p < 0.05). Higher levels in mRNA for the node/notochord markers *FOXA2*, *SHH*, and *Noggin* observed at 3–5 days (D3–D5) after induction of differentiation as compared to culture in basal conditions (*p < 0.05). **b** Notochord marker *NOTO* was not upregulated at any time following induction of differentiation with FGF2 and BMP4; however, addition of Wnt-3a and Activin A promoted an early (D2) and sustained elevation (D3–D5) in mRNA for *NOTO* (*p < 0.05). This observation was key in our choosing to supplement colony cultures with Wnt-3a and Activin A at the earliest time points, days 1–3 (D1–D3). bFGF2 basic fibroblast growth factor, BMP bone morphogenetic protein 4, MIXL1 paired-type homeobox transcription factor identified in human, CDX2 member of the caudal-related homeobox transcription factor family, FOXA forkhead box protein A2, SHH sonic hedgehog, NOG noggin, NOTO notochord homeobox

We first evaluated mRNA expression in EBs at an early stage (1–5 days) of mesodermal differentiation induced by BMP4 and FGF2. RT-PCR analysis showed that highest expression level of *T* and *MIXL1* were achieved at early stages (day 2) of iPSC differentiation, whereas the highest expression of other mesoderm markers, *CDX2* and *FOXA2*, was observed at day 3 (Fig. 3a). Throughout, we examined the expression of expected markers based on their relation to the mesodermal lineage; we did not examine expression of markers consistent with the ectodermal or endodermal lineages, nor did we evaluate the loss of pluripotency markers, which was a limitation of our approach. We additionally examined expression of signaling molecules previously shown to be important in NP differentiation, including the node/notochord markers *SHH* and *NOG*. We did find significant increases in mRNA for *SHH* and *NOG* at 3–5 days (Fig. 3b). It is known that notochord homeobox (NOTO) acts downstream of both FOXA2 and T [35, 36], and is necessary for notochord development. However, BMP4 and FGF2 supplementation did not induce expression of *NOTO* higher than the levels measured in basal medium (Fig. 3b). As NP cells are specified in the node by Wnt and Nodal (activin) signals at the gastrula stage, Wnt-3a and Activin A were added to promote elevated expression of *NOTO*. Thus, we selected BMP4, FGF2, Wnt-3a, and Activin A as growth factors to differentiate hiPSCs into NP-like cells and defined a temporally varying differentiation protocol called NP differentiation media (NPDM) (Fig. 1 and Table 1).

### Development of NP-like cells through multistage iPSC differentiation

**Fig. 4:**
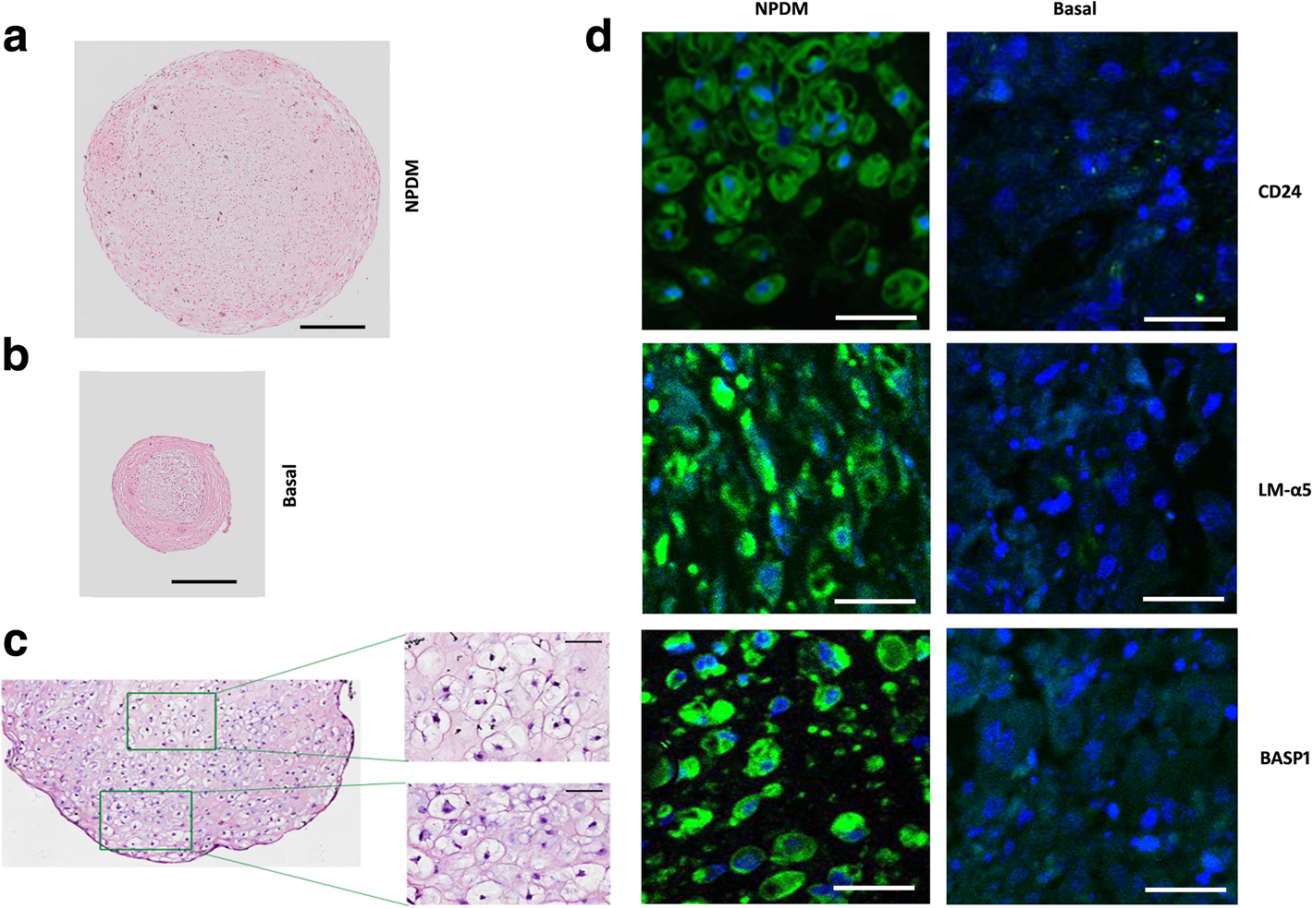
Images of hiPSC cultured for 36 days with nucleus pulposus differentiation media (NPDM) or basal media. **a** H&E staining of pellets cultured in NPDM (scale bar = 400 μM) or (**b**) basal medium showing formation of larger pellets with a vacuolated morphology when hiPSCs were promoted to differentiation in NPDM (scale bar = 400 μM). (**c**) When cultured in NPDM, human iPSCs contained vacuole-like structures (scale bar = 50 μm). **d** NPDM promoted expression of CD24, LM-α5, and BASP1 protein by 28 days of culture (scale bar = 50 μm). LMα5 alpha-5 subunit of heterotrimeric laminin, BASP1 brain abundant membrane attached signal protein 1

The NPDM protocol defined earlier was used to support the differentiation of iPSCs from an initial 7 days in a colony, followed by 7 days in monolayer culture plus an additional 21-day pellet culture. iPSCs promoted to differentiate under the NPDM protocol led to the formation of larger, more uniform pellets that contained cells with the vacuole-like structure characteristic of NP-like cells (Fig. 4a, c), as compared to iPSCs cultured in basal media that developed no vacuoles (Fig. 4b). On average, iPSCs differentiated under the NPDM protocol formed pellets of 1.9 mm diameter (*n* = 8) as compared to 0.51 mm for cells in basal media (*n* = 8). Immunohistochemistry analysis showed that cells following this NPDM stained positive for expression of NP markers including CD24, LM-a5, and BASP1, as compared to no staining in control cells cultured in basal medium (Fig. 4d).

### Enrichment of T-positive cells during mesodermal lineage differentiation

**Fig. 5:**
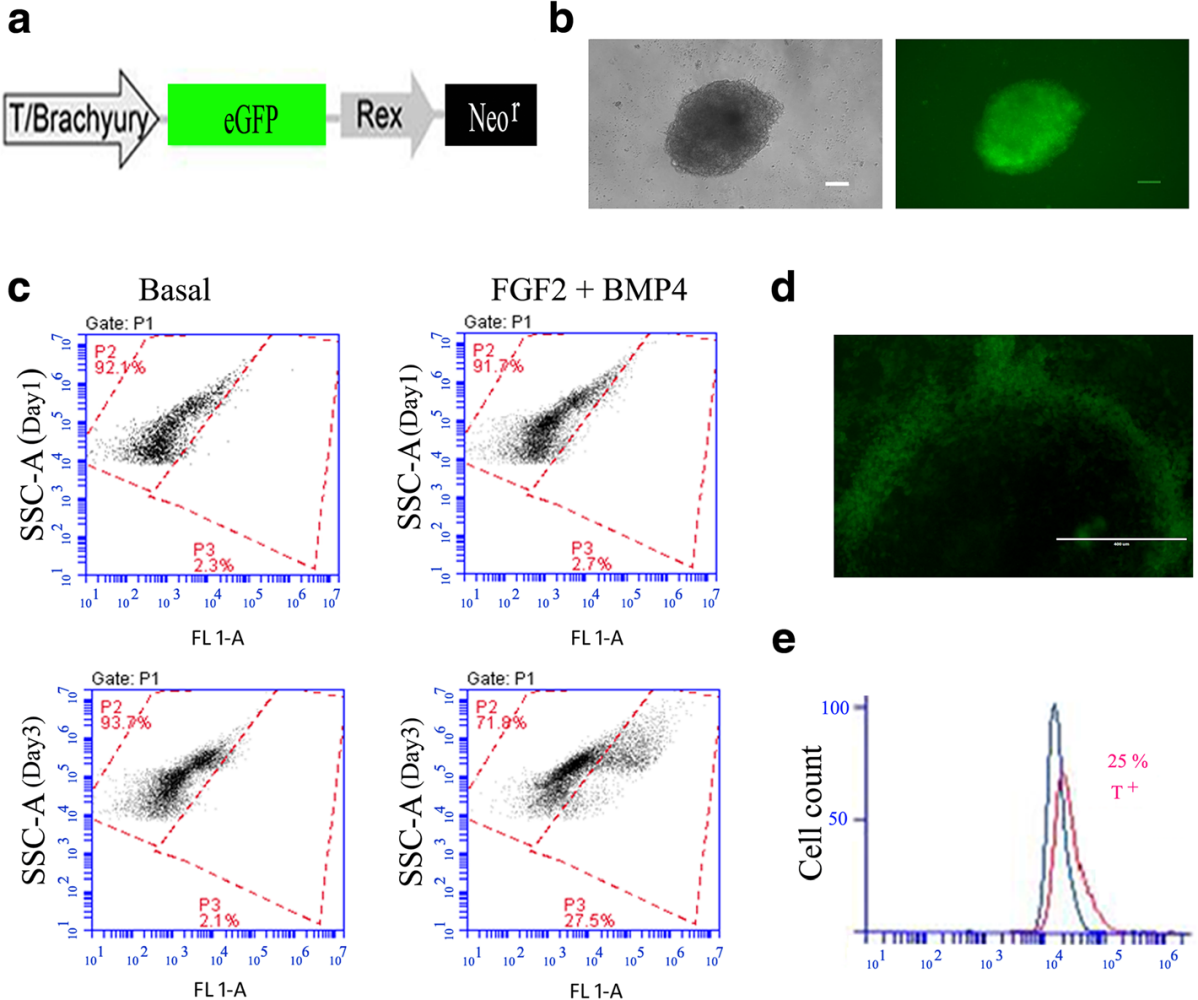
Characterization of notochordal transcription factor brachyury expression via the T-fluorescent reporter construct. **a** Cells transfected with lentivirus containing plasmid for eGFP downstream of the brachyury (T) promoter. **b** Bright-field image and fluorescent image of a differentiated cell colony following culture in basal medium when supplemented with 40 ng/ml BMP4 and 20 ng/ml FGF2 (3 days) (scale bar = 100 μM). **c** eGFP expression detected via flow cytometry after supplementation with BMP4 and FGF2 at day 3 (showing 27.5% of cells for this population). **d** Before cell sorting, LV-T-GFP-positive cells were localized around the periphery of a differentiated cell colony (scale bar = 400 μM). **e** When cultured in basal medium supplemented with BMP4 and FGF2, flow cytometry analysis showed 25.4% of cells to be positive for T-GFP^+^ in differentiated hiPSCs. GFP green fluorescent protein, bFGF2 basic fibroblast growth factor, BMP bone morphogenetic protein 4

As brachyury (T) is one of the earliest transcriptional factors involved in mesoderm development, we sought to enrich for cells that expressed this marker of mesodermal lineage differentiation and use them for NP differentiation. Undifferentiated hiPSCs were transduced with a lentiviral plasmid containing an eGFP reporter downstream of the T promoter (Fig. 5a). Expression of eGFP in the hiPSC reporter cell line was observed when cells were cultured in colonies for 3 days post mesodermal lineage induction with FGF2 and BMP4 (Fig. 5b). While an average of 22.5% of cells (*n* = 3) at day 3 were GFP positive via flow cytometry (Fig. 5c), it appeared that the majority of the GFP^+^ cells were localized in the periphery of the cell (Fig. 5d). Approximately 25% of all cells were positive for brachyury protein under these conditions via flow cytometry (Fig. 5e). These results indicated that mesoderm lineage cells can be induced by combined application of BMP4 and other cytokines, and GFP expression can be a reliable reporter of brachyury transcription during induced differentiation.

### Differentiation of FACS GFP^+^ iPSCs

To investigate the potential for an enriched T-GFP^+^ cell population to promote NP cell phenotypes, transduced hiPSCs were differentiated according to the NPDM protocol as described and compared against those promoted to differentiation in the T-GFP^−^ cell population. Immunocytofluorescence showed the positive expression of CD24 and CD239 in these sorted T-GFP^+^ cells when undergoing differentiation for 12 days as described in step 1b and step 2 of the NPDM protocol (Additional file 2), showing that the GFP^+^ cells were uniform and stable as they underwent differentiation toward an NP-like phenotype.

**Fig. 6:**
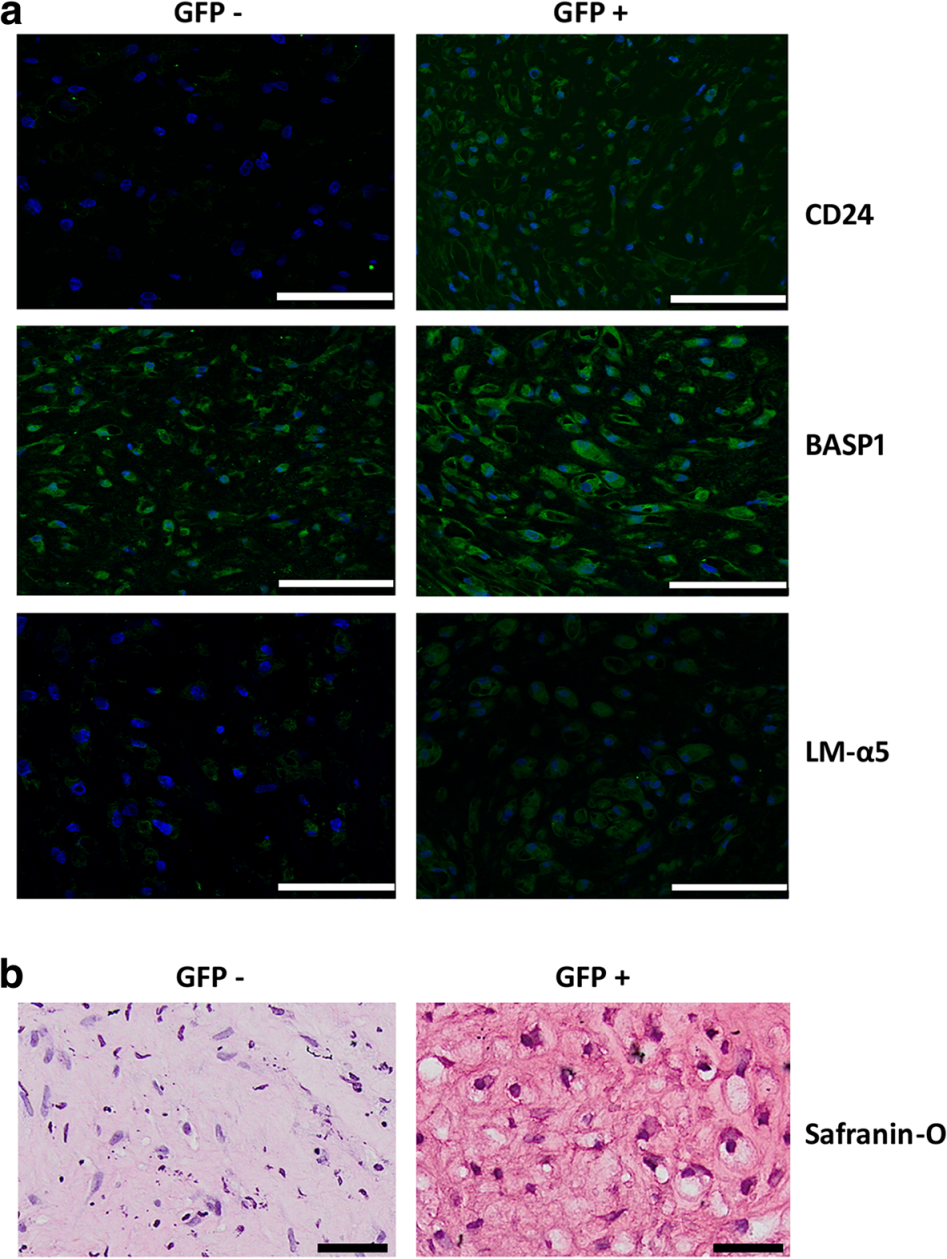
NP markers enriched when differentiating human iPSCs sorted for T^+^ expression. **a** Immunohistochemistry showing higher expression of CD24, BASP1, and LMα5 of T-GFP^+^ sorted cells when cultured in NPDM as pellets (scale bar = 100 μM). **b** Safranin-O staining showing higher expression of GAGs in sorted T-GFP^+^ cells of pellets cultured in NPDM (scale bar = 50 μM). GFP green fluorescent protein, BASP1 brain abundant membrane attached signal protein 1, LMα5 alpha-5 subunit of heterotrimeric laminin

At the end of pellet culture for these GFP^+^ cells, immunofluorescence showed higher and spatially uniform expression of NP markers CD24, BASP1, and LMα5 in sorted T-GFP^+^ cell pellets as compared to sorted T-GFP^−^ cell pellets (Fig. 6a). Higher and more spatially uniform expression of GAG was also observed in GFP^+^ pellets (Fig. 6b). Pellet cultures containing enriched GFP^+^ cells did not appear to be elevated in expression of the vacuolated cell morphology, although markers of the NP cell vacuole have not yet been defined.

## Discussion

In this study, human iPSCs were successfully differentiated into NP-like cells using a defined differentiation protocol, and further enriched using fluorescence sorting with a GFP–brachyury reporter system. Although the temporal involvement of transcriptional factors (TFs) in the development of the notochord and the postnatal NP cell remains unclear, three major TFs involved in the process for notochord specification and differentiation have been identified: Forkhead A2 (FOXA2), Brachyury (T), and Notochord homeobox (Noto), as demonstrated across many species including human [5–10]. In stage I (days 1–7) of our chemically defined differentiation medium, expression of T and FOXA2 was activated by the presence of BMP4 and FGF2, but expression of Noto was absent without supplementation with Activin A and Wnt-3a (Fig. 3). Thus, we adopted a protocol with early-stage exposure to Activin A and Wnt-3a, followed by a more prolonged but temporally varying exposure to BMP4 and FGF2 that was also necessary for promotion of the mesodermal lineage. Using this multistage protocol, iPSCs were differentiated to an NP-like phenotype, showing vacuolated cell morphology and expression of NP markers including CD24 and elevated glycosaminoglycan expression via Safranin-O staining. Protein levels of the LMα5 subunit and BASP1 were also identified; while BASP1 was originally considered to be a “secondary” marker of the NP cell phenotype for the need to identify in human NP at the protein and mRNA level, BASP1 has recently been identified in juvenile human NP tissues [33] and also in the differentiated human iPSCs of a recent study [37] which is consistent with the definition of a primary NP marker.

CD24 expression is a marker of unique interest in the NP-cell lineage, as CD24 expression has been found in juvenile NP cells but not in anulus fibrosus or older disc cells [33, 38, 39]. Indeed, CD24 is a widely used NP marker that has been used in our prior work to select NP-genic progenitors from mouse iPSCs [22] and it may serve more broadly as a molecular marker of progenitor cells in the NP [4]. Expression of CD24 has become the ‘gold standard’ for illustrating successful NP cell differentiation [33, 37] although it is a marker of multiple cell types including epithelial cells [40–42]. We could benefit from further work testing for expression of alternate phenotypic markers to confirm that CD24 is indeed requisite for defining a terminally differentiated stem cell as an NP cell.

Enrichment for brachyury-expressing cells using a GFP reporter showed further enhancement of cell differentiation to the NP cell lineage. Sakai and coworkers have suggested that Tie2 and GD2 expression are important cell-associated markers upstream of CD24 expression in NP progenitor cells [43, 44], and that each of these markers may be used to sort cells for the NP progenitor populations in human iPSCs. Here we showed successful upregulation of NP markers for hiPSCs when they were sorted for T^+^ expression prior to further steps of differentiation. Our ultimate goal was to define a temporal culture protocol that could promote NP-genic progenitors from iPSCs with or without cell sorting as a necessary step in the differentiation protocol. In addition to evaluating the potential for alternate cell selection strategies, future work can further optimize the application of defined soluble mediators to the enriched T-GFP^+^ cell population studied here**.**

Prior studies have shown a role for environmental conditions, such as stiffness and ligand presentation, in regulating progenitor and stem cell differentiation into multiple cell phenotypes [45]. Our prior work has demonstrated that both soft substrates and laminin-functionalized surfaces are important for promoting maintenance of expression of NP-associated molecular markers [34, 46–49]. Other studies have demonstrated that culture of progenitor cells in media supplemented with notochordally derived conditioned media may be important regulators of NP cell differentiation and NP cell phenotype [14, 50–52]. Here, we used serum-free culture media that were partly informed by our prior work with inducing mouse iPSCs to express NP markers, but with the addition of supplements to enhance collagen and proteoglycan production as has been seen in chondrogenic cultures. These conditions may have promoted the expression of chondrocyte markers that were not studied here, such as select collagen isoforms and integrin subunits [53–55]. Further work would be needed to confirm and validate this media supplement as appropriate for NP-genic, but not chondrogenic, differentiation conditions. Further work is also needed to better understand the role of cell morphology, such as vacuoles and cell size, and markers of the NP cell phenotype, in order to optimize strategies to better differentiate iPSCs into stable NP cells that can be used for therapeutic purposes. From this study, we can conclude that a defined protocol of media supplementation informed by known notochordal and NP cell development has value in promoting iPSC differentiation into NP-like cells *in vitro.*

Cell-based IVD therapy has emerged as an area of enormous need but with limited demonstrations of proof of concept, with studies reporting matrix regenerative potential for many cell sources, including autologous (allogeneic) chondrocytes, primary IVD cells, and stem cells [4, 38, 56–62]. The question of cell source is vital for cell-based IVD regeneration, given that the availability of autologous NP cells is extremely low in the adult, and that the adult cell phenotype differs from that of the younger NP cell. In early work, autologous or allogeneic NP cells were isolated, expanded, and reimplanted at high density in the IVD in animal studies, demonstrating some beneficial effects in inhibiting some degenerative changes such as loss of disc height with nucleotomy [19, 21, 63–66]. Autologous disc cell transplantation has also been evaluated in clinical trials for follow-up treatment to discectomy [67, 68], leading to the emergence of clinical products and platforms that support autologous cell supplementation to the IVD. Allogeneic chondrocytes [69] have also been tested in clinical trials for delivery to the disc space. Given the limited availability of healthy, autologous IVD cells, there has been great interest in using stem cells for disc cell supplementation, including mesenchymal stem cells (MSCs) from bone marrow [70, 71], adult adipose tissue [72], and umbilical cord matrix [23, 73–75]. Furthermore, the availability of large numbers of genetically defined NP cells may allow screening for disease-modifying drugs as therapeutics for inhibiting degeneration or inducing regeneration of the NP [76]. Our use of hiPSCs here points toward the potential for an adult cell source that could be used to address the therapeutic needs for a pathology of broad magnitude, such as degenerative disc disease, and motivates further studies with potential to translate to clinical utility.

## Conclusions

A defined protocol of media supplementation informed by known notochordal and NP cell development has been demonstrated to promote iPSC differentiation into NP-like cells *in vitro*, with potential applications for regeneration of the IVD.

## Additional files

Additional file 1: **Table S1.** Presenting source of probe/primers for real-time PCR (18s RNA is control). (DOCX 14 kb)

Additional file 2: **Figure S1.** Showing immunofluorescence of sorted T-GFP^+^ cells promoted to differentiate in culture. (A) Immunocytofluorescence showed the positive expression of CD24 (green) and (B) positive expression of CD239 (red) in sorted T-GFP^+^ cells when differentiated in monolayer for 12 days using step 1b and step 2 of NPDM protocol. (C) Yellow denotes colocalization of the markers. All cells counterstained with DAPI. Scale bar = 20 μm. (DOCX 995 kb)

## Abbreviations

BASP1: Brain abundant membrane attached signal protein 1
BMP: Bone morphogenetic protein 4
CD239: Lutheran blood group glycoprotein
CD24: Cluster of differentiation 24 protein
CDX2: Member of the caudal-related homeobox transcription factor family
EB: Embryoid body
FGF2: Basic fibroblast growth factor
FOXA2: Forkhead box protein A2
GDF5: Growth differentiation factor 5
GFP: Green fluorescent protein
hiPSC: Human induced pluripotent stem cell
IVD: Intervertebral disc
LMα5: Alpha-5 subunit of heterotrimeric laminin
MIXL1: Paired-type homeobox transcription factor identified in human
MSC: Mesenchymal stem cell
NOG: Noggin
NOTO: Notochord homeobox
NP: Nucleus pulposus
NPDM: Nucleus pulposus differentiation media
rRNA: Ribosomal RNA
SHH: Sonic hedgehog
T: Brachyury
TGβ3: Transforming growth factor beta-3
