# Supplementary material for: Differentiation of human induced pluripotent stem cells into nucleus pulposus-like cells
Source: Stem Cell Res Ther. 2018 Mar 9;9:61. doi: 10.1186/s13287-018-0797-1 (PMC5845143; doi:10.1186/s13287-018-0797-1)
Supplement: Supplementary file 1 — Table S1. Presenting source of probe/primers for real-time PCR (18s RNA is control). (DOCX 14 kb) [file 13287_2018_797_MOESM1_ESM.docx]

**Additional file 1: Table S1: Source of probe/primers for real-time PCR, 18s RNA is control**

| T | Hs00610080_m1 |
| --- | --- |
| MIXL1 | Hs00430824_g1 |
| CDX2 | Hs00230919_m1 |
| FOXA2 | Hs00232764_m1 |
| SHH | Hs00179843_m1 |
| NOTO | Hs01377437_m1 |
| NOG | Hs00271352_s1 |
