# Supplementary material for: Differentiation of human induced pluripotent stem cells into nucleus pulposus-like cells
Source: Stem Cell Res Ther. 2018 Mar 9;9:61. doi: 10.1186/s13287-018-0797-1 (PMC5845143; doi:10.1186/s13287-018-0797-1)
Supplement: Supplementary file 2 — Figure S1. Showing immunofluorescence of sorted T-GFP+ cells promoted to differentiate in culture. (A) Immunocytofluorescence showed the positive expression of CD24 (green) and (B) positive expression of CD239 (red) in sorted T-GFP+ cells when differentiated in monolayer for 12 days using step 1b and step 2 of NPDM protocol. (C) Yellow denotes colocalization of the markers. All cells counterstained with DAPI. Scale bar = 20 μm. (DOCX 995 kb) [file 13287_2018_797_MOESM2_ESM.docx]

**
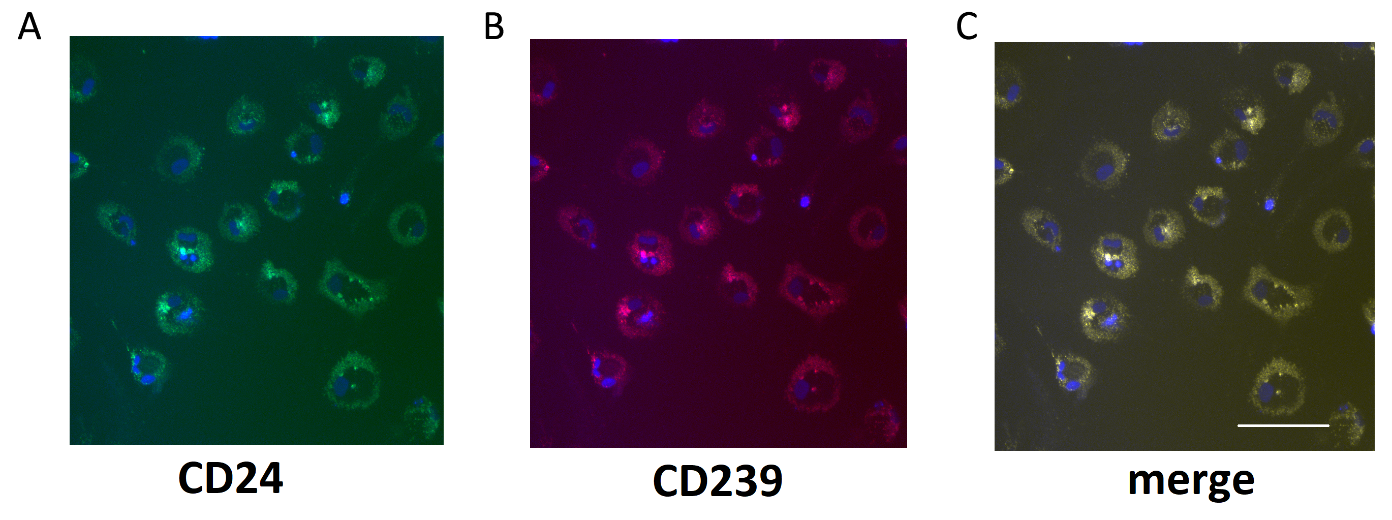
**

**Additioal file 2: Figure S1.** (A) Immunocytofluorescence showed the positive expression of CD24 (green) and (B) positive expression of CD239 (red) in sorted T-GFP+ positive cells when differentiated in monolayer for 12 days using the protocol described in step 1b and step 2 of NPDM protocol. (C) Yellow denotes co-localization of the markers. All cells are counter-stained with DAPI. Scale bar = 20 microns.
